# Case Report of Very-Low-Dose Fentanyl Causing Fentanyl-Induced Chest Wall Rigidity

**DOI:** 10.7759/cureus.43788

**Published:** 2023-08-20

**Authors:** Ronza Zoorob, Logan Uptegrove, Benjamin L Park

**Affiliations:** 1 Anesthesia, Lake Erie College of Osteopathic Medicine, Indiana, USA; 2 Anesthesia, Indiana Regional Medical Center, Indiana, USA

**Keywords:** fentanyl-induced chest wall rigidity, opioid-induced chest wall rigidity, opioid-induced apnea, opiates, wooden chest syndrome

## Abstract

Wooden chest syndrome (WCS) is a rare phenomenon of opioid-induced skeletal muscle rigidity causing respiratory failure and inability to ventilate. The most common opioid associated with WCS is the synthetic opioid fentanyl. Fentanyl has been called the deadliest drug in America. With the use of fentanyl in critical care units and operation rooms, it is important to better understand fentanyl’s side effects and predisposing factors of WCS. The symptoms of WCS are often seen in lower fentanyl doses than what would cause apnea. In this case report, we present a case of WCS with an extremely low dose of fentanyl, i.e., 50 μcg (0.49 μcg/kg), in an 80-year-old patient with a medical history significant for chronic inflammatory demyelinating polyneuropathy (CIDP) and Guillain-Barré syndrome (GBS).

## Introduction

Wooden chest syndrome (WCS), also known as fentanyl-induced rigidity, is a rare but known complication of intravenous (IV) fentanyl administration. The incidence of WCS is unknown due to multiple factors, including mortality in untreated patients and incident locations being unmonitored. Fentanyl is a synthetic opioid that is typically used as an analgesic medication. It is often used in conjunction with sedatives in procedural settings. Historically, WCS is associated with rapid infusion, high dose, or extremes of age [1,2]. With the current opioid epidemic and the increase in opioid use for long-term sedation and intubation, there is a higher incidence of providers encountering WCS. In this case report, we describe the case of a patient who was administered an extremely low-dose fentanyl and developed fentanyl-induced chest wall rigidity. This patient received only 50 μcg (0.49 μcg/kg) of fentanyl, for anxiolysis, preoperatively, which is significantly lower than the dose usually associated with WCS.

## Case presentation

An 80-year-old male with a history of hypertension (HTN), gastroesophageal reflux disease (GERD), atrial fibrillation (AFib), and chronic inflammatory demyelinating polyneuropathy (CIDP) and Guillain-Barré syndrome (GBS), presented to the emergency department with a left arm swelling. The patient was on apixaban and monthly IV immunoglobulin (IVIG) infusions. The patient had a mediport in his left jugular vein and had been taking oral anticoagulation for many months with no issues. The mediport was placed to facilitate the monthly IVIG infusions for the treatment of CIPD. The patient was severely limited and weakened by GBS and CIDP. He was able to walk a maximum distance of 80 feet with two-person assistance. Preoperative vitals found the patient mildly tachycardic with a heart rate of 106 bpm, a blood pressure of 107/74 mmHg, a respiratory rate of 10 breaths per minute, and a pulse oximetry of 96% on 2L oxygen via nasal canula. The patient had no pain and IV access of a 22G IV in the right hand and a 18G IV in the right antecubital fossa for medication administration.

With the presenting symptom of a unilaterally swollen left upper arm and a medical port on the same side, an upper-extremity ultrasound was performed. The ultrasound demonstrated a deep vein thrombosis (DVT) of the left subclavian vein. The patient was started on an IV heparin drip in the emergency department and was admitted to the hospital. Upon consulting with vascular surgery, it was deemed favorable to remove the port as it was the nidus of the DVT.

The patient was seen by the anesthesia team and designated an American Society of Anesthesiologists (ASA) IIIE score. The anesthetic plan for the patient was monitored anesthesia care (MAC). The anesthesia team assessed the patient preoperatively, and he was administered 50 μcg of fentanyl for analgesia and anxiolysis immediately prior to transport to the operating room. The patient remained in verbal communication with the anesthesia team and no other monitors were used during transport, until arriving at the operating room. As the patient was being brought into the operating room, the patient stopped conversing and displayed complete body rigidity with arms stiffened. The patient also became apneic at that time. The patient’s posturing and apnea occurred two to three minutes following the administration of fentanyl.

A pulse oximeter was rapidly applied, and the patient was found to be hypoxic with a pulse oximetry reading of 70% and decreasing rapidly. At this juncture, additional assistance was requested, while further monitors were placed, and supplemental oxygen was administered to the patient. The patient continued to stiffen, reaching a very rigid state, preventing an oral airway from being inserted into the patient’s mouth. At this moment, additional assistance arrived, and the patient was administered 70 mg bolus of propofol along with two-handed mask ventilation. Shortly after, the patient appeared to relax, and assisted bag-valve-mask ventilation was initiated. As the patient resumed spontaneous ventilation, a decision to place a laryngeal mask airway (LMA) to support ventilation was made, and an LMA size 4 was atraumatically placed. The initial end tidal carbon dioxide was 65 mmHg but started to descend shortly after, with hand-assisted ventilations. The surgery was performed rapidly, with minimal blood loss, and the LMA was removed without any complications.

The patient was transported to the post-anesthesia care unit (PACU) for recovery. When the patient was questioned about recollection of the incident and what had transpired, he stated that he had no recollection of any events that occurred and the last memory the patient had was traveling down the operating room hallway and then emerging in the PACU. The patient recovered without any further incident.

## Discussion

Fentanyl was initially synthesized in 1959 by Janssen Pharmaceuticals. After the synthesis, it took several years for fentanyl to make a significant difference in clinical practice [1]. The potency of fentanyl has demonstrated a significantly lower therapeutic window than other commonly used opiates [2]. This is supported by various research sources, which revealed that most opiate overdoses and fatalities involve fentanyl and other synthetic opiates. Although fentanyl is relatively cardiovascular stable, it can cause bradycardia and hypotension depending on the patient’s past medical history and dosing. Respiratory depression, with decreased minute ventilation, and decreased response to hypercarbia and hypoxia are also common. Pruritus and nausea are also common side effects of opioids. In 2018, fentanyl was labeled as the deadliest drug in the United States [1]. 

A seemingly uncommon but known side effect of administration of IV opiates is WCS, also known more modernly as opioid-induced chest wall rigidity or specifically fentanyl-induced chest wall rigidity [3]. Common predisposing factors for the development of rigidity includes extremes of age and high doses of fentanyl. The two most common factors associated with the development of WCS includes opiate lipophilicity and the speed of injection [1,4]. WCS was first described in 1953 and associated with opiates, such as morphine, meperidine, and methadone [5]. The description of WCS occurred with other opiates before the synthesis of fentanyl; however, fentanyl depicts a larger percentage of WCS cases compared to prior opiates, likely because of the high lipophilicity of fentanyl [2]. In 2018, Trujillo and colleagues reviewed cases from 2014 to 2017 at a level 2 trauma center and objectively described ventilator parameters and opioid dosage. Of the eight cases they reviewed, it seems likely that many more cases went undiagnosed and that WCS is an underdiagnosed complication of opiates [6]. This is supported by evidence from Kinshella et al. who investigated overdoses at a Canadian-supervised injection site and described typical and atypical overdose presentations [7]. These observations suggest that muscle rigidity may be a significant cause, and under-reported cause, of death in opiate overdose deaths. When a cardiac arrest occurs in a patient with WCS, cardiopulmonary resuscitation (CPR) with chest compressions can be difficult to near impossible. This is supported by emergency room survival rates that are significantly lower for fentanyl compared to heroin [8].

Muscle rigidity was the most common symptom of atypical opiate overdoses. Muscle rigidity occurred in 48% of the atypical overdose manifestations, but muscle rigidity was only observed in 15% of total overdoses at the time. These overdoses occurred with the opiates morphine and heroin [7]. WCS commonly occurs within minutes of administration and lasts on average 10-12 minutes [2]. The typical treatment for WCS is to control the airway and respirations while administering a neuromuscular blocking agent. 

The pathophysiology of WCS appears to be a centrally mediated phenomenon. This is supported by evidence that fentanyl is found on the autopsy of many sudden overdose deaths and the absence of its metabolite, norfentanyl [9]. There are many mu receptors found in the locus coeruleus (LC) of the brainstem. The LC is found in the rostral pons along the lateral floor of the fourth ventricle, between the midbrain and medulla oblongata. The LC is the main area for the development of norepinephrine (NE) found in the brain [1]. Multiple studies reveal that the rigidity of respiratory muscles induced by fentanyl is mediated by the agonism of mu-opioid receptors in the LC, which increases noradrenergic outflow from the LC by activating α1-adrenergic receptors in the LC and spinal cord. When fentanyl is injected directly into the LC, skeletal muscle rigidity occurs, suggesting coerulospinal noradrenergic involvement [10]. Ablation of the LC or separation from the spinal cord was shown to abate this muscle rigidity [1]. This NE outflow from the LC to the spinal cord is the initial part of the proposed mechanism of WCS, although the synthesis of NE is seen to be reduced over the long term with opiates [11]. In addition, early reports of WCS, prior to fentanyl, described antagonism with naloxone as a typical remedy, further supporting a central mechanism [6]. Interestingly, there is a single case report of WCS occurring after naloxone use, being used for an opioid overdose [12]. 

WCS signs and symptoms include a drop in oxygen concentration on pulse oximetry, muscles stiffening up after fentanyl administration, and breath-holding spells due to chest wall contraction, causing elevated airway pressures. Muscle rigidity increases oxygen consumption leading to a faster than apnea alone desaturation of pulse oximetry. Bag-valve-mask ventilation is usually implemented to overturn the hypoxia, but only after administering a neuromuscular blocking agent is it successful, as bag-valve-mask ventilation is often faced by high resistance due to chest wall stiffness [5]. Neuromuscular blocking agents work as competitive antagonists of nicotinic receptors, which decrease skeletal muscle contraction. Hypertension is also another manifestation of WCS, likely related to catecholamine release [1]. 

Multiple studies have demonstrated that fentanyl-induced wall rigidity is mainly affected by the dosing of fentanyl and age of the recipient. Typically, dosing in WCS requires higher doses than seen in this case. Moreover, patients over the age of 60 are more prone to WCS, an additional risk factor seen in the case presented [13].

There is also a known link between WCS and central neurologic diseases, such as essential tremor or Parkinson's disease [13]. While this patient does not have a known centrally acting neurologic disease, he does have a history of CIDP, a peripheral demyelinating disease known to affect both proximal and distal peripheral nerve fibers [14]. CIDP has been linked to vaccines and most recently the COVID-19 mRNA vaccines [15]. If CIDP were linked to WCS, this would be an important causality for providers to be aware of. This poses the question of whether and how a peripheral demyelinating condition can be an influencing factor for causing skeletal muscle rigidity, a topic worth looking into.

## Conclusions

WCS is a result of activating mu opioid receptors in the central nervous system through a dopaminergic pathway, resulting in skeletal muscle rigidity. Reversing WCS is accomplished by discontinuing the offending agent, fentanyl in this case, and administering neuromuscular blocking agents. With the surge of fentanyl use in critical care and procedural settings, the incidence of fentanyl-induced rigidity phenomenon is higher and should be further explored. This patient experienced WCS with a dose of fentanyl much lower than reported previously. In addition, this patient was supportively managed without the need for a neuromuscular blocking agent, which is uncommon for the management of WCS.
